# Complete genome sequence of *Deinococcus rubellus* Ant6 isolated from the fish muscle in the Antarctic Ocean

**DOI:** 10.3389/fbioe.2023.1257705

**Published:** 2023-10-16

**Authors:** Surajit De Mandal, Sathiyaraj Srinivasan, Junhyun Jeon

**Affiliations:** ^1^ Department of Biotechnology, Yeungnam University, Gyeongsan, Republic of Korea; ^2^ Department of Bio and Environmental Technology, College of Natural Science, Seoul Women’s University, Seoul, Republic of Korea; ^3^ Plant Immunity Research Center, Seoul National University, Seoul, Republic of Korea

**Keywords:** *Deinococcus*, Antarctic Ocean, radioresistance, cell factory, complete genome

## Introduction

The Antarctic Ocean is considered one of the harshest environments, with nutrient limitation, extremely low temperatures, limited gas exchange, and high ultraviolet (UV) radiation, which triggers the generation of reactive oxygen species (superoxide anions, hydrogen peroxide, highly reactive hydroxyl radicals), resulting in oxidative damage to the cellular macromolecules. Species adapted to such extreme habitats are known to be equipped with novel defense mechanisms to prevent oxidative damage either by non-enzymatic or enzymatic processes. It has also been reported that low temperature reduces the intracellular diffusion of free radicals in the organism, thereby increasing resistance to radiation (Sommers et al., 2002; Dartnell et al., 2010).

The bacterial members under the family *Deinococcaceae* are well known for their unique radiation-resistance characteristics and have been isolated from a variety of environments, including soil, air, feces, water, hot springs, irradiated food, and fish muscle (Blasius et al., 2008). Although the mechanism of extreme radioresistance in *Deinococcus* has not been fully elucidated, it has been suggested that extreme radioresistance results from a combination of strategies, including the use of efficient DNA repair pathways, protection of proteins from oxidation, and a condensed nucleoid that limits the dispersion of genome fragments after irradiation (Slade et al., 2009; Daly et al., 2010; Floc’h et al., 2019). The genome of *Deinococcus radiodurans* encodes a large number of Ser/Thr protein kinases (STPKs) and guanine quadruplex (G4) DNA structure dynamics, many of which have been shown to participate in DNA damage response (Rajpurohit and Misra, 2010; Beaume et al., 2013; Rajpurohit et al., 2016; Maurya et al., 2018). Another theory suggests that the accumulation of manganese complexes inhibits the production of iron-dependent reactive oxygen species or forms antioxidant complexes with minor metabolites in cells, thereby protecting the proteome from ionizing radiation (IR) damage (Daly, 2009). They have also been reported to utilize different carbon sources and express novel engineered functions which makes them a suitable candidate for various biotechnological applications such as the decomposition of toxic compounds, bioremediation, and production of industrial and medically important molecules (Gerber et al., 2015).

A new bacterial strain, *Deinococcus rubellus* Ant6, was isolated from fish muscle in the Antarctic Ocean and showed a high gamma radioresistant activity (D10 value of 4 kGy) (Choi et al., 2016) (Supplementary Table S1). Therefore, in this study, we sequenced and annotated the complete genome of Ant6 using PacBio sequencing and highlights the genomic clues behind its survival mechanisms in extreme Antarctic environments, including the antioxidative systems. The complete genomic information will be highly useful to engineer the Ant6 strain and use it as a cell factory to produce biotechnological important molecules.

## Materials and methods

### Genomic DNA isolation

Strain Ant6 of *D. rubellus* was cultured for 24 h in R2A media and the genomic DNA was extracted using the Wizard Genomic DNA extraction kit (Cat # *A1120*, *Promega* Corp., Madison, WI, United States) following the manufacturer’s instructions. The quality and quantity of purified genomic DNA were determined by Nanodrop and agarose gel electrophoresis.

### Genome sequencing, annotation, and analysis

The genome of the strain Ant6 was sequenced by the Pacific Bio-sciences RS II platform, and a library was constructed according to the manufacturer’s recommendations. The PacBio long reads were assembled using the HiCANU v. 2.1 assembler (Nurk et al., 2020). The Prokaryotic Genome Annotation Pipeline (PGAP) was used for the annotation of the Ant6 genome. The tandem repeats and the CRISPR arrays were predicted using the Tandem Repeat Finder (Benson, 1999) and CRISPRFinder pipeline (Grissa et al., 2007), respectively. A circular representation of the chromosome of *D. rubellus* Ant6 was generated using the CGViewer Server (Grant and Stothard, 2008). Prediction of protein domains were carried out using SMART (http://smart.embl-heidelberg.de/), CDD (https://www.ncbi.nlm.nih.gov/Structure/cdd/wrpsb.cgi), and InterProScan program (https://www.ebi.ac.uk/interpro/). The Codon Tree pipeline of BV-BRC was applied to generate phylogenetic relationship of Ant6 with the closely related species (Olson et al., 2023). Average nucleotide identities (ANI) were analysed based on BLAST + ANIb using the JSpecies web server (Richter et al., 2016). The DNA-DNA hybridization (DDH) values was calculated using Genome-to-Genome Distance Calculator (GGDC) (http://ggdc.dsmz.de/ggdc.php#) (Meier-Kolthoff et al., 2013).

*D. rubellus* Ant6 and another two bacterial genomes (*D. radiodurans* R1 and *Escherichia coli* K12) from NCBI databases were used for the comparative genome analysis. The Gene functional categories were identified based on the Clusters of Orthologous Groups (COGs) using Eggnog Mapper (Huerta-Cepas et al., 2017). Putative orthologous genes among the three strains were determined by reciprocal best hits with a minimum of 30% identity. Heatmap was constructed using CIMMiner (https://discover.nci.nih.gov/cimminer/oneMatrix.do).

## Interpretation of data set

### General genome features

**FIGURE 1:**
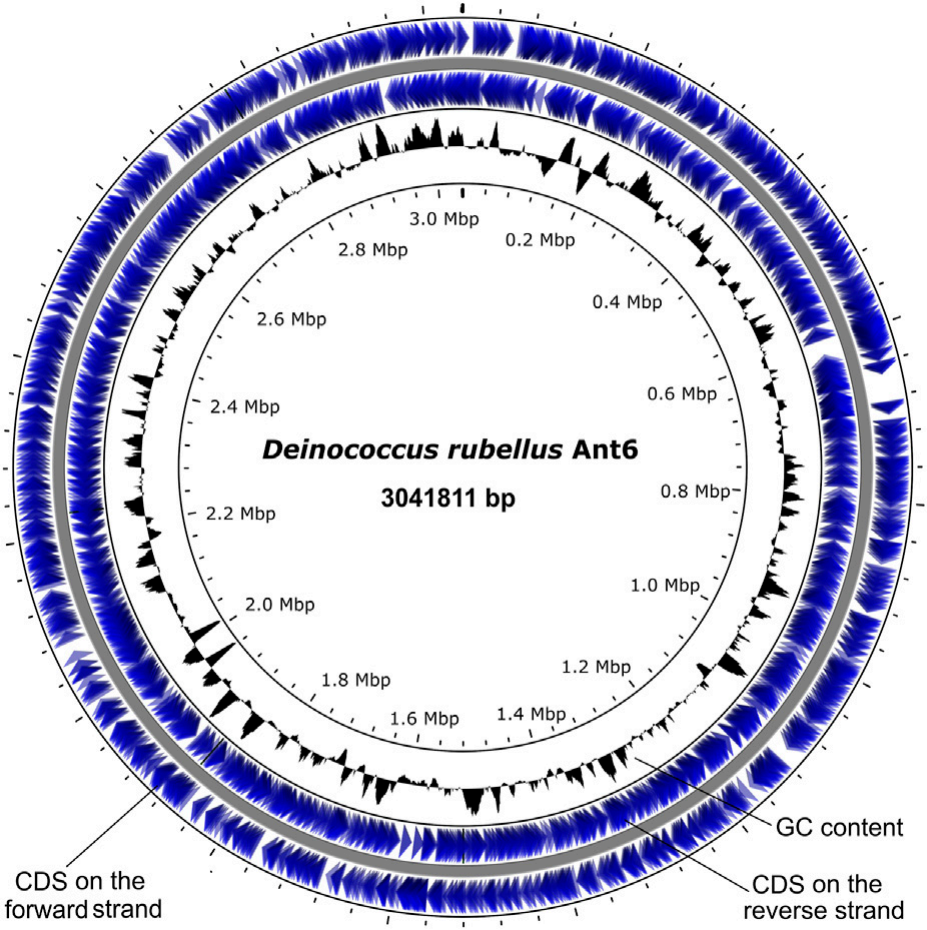
Circular representation of the chromosome of *Deinococcus rubellus* Ant6.

The genome of *D. rubellus* Ant6 has a 3041811 bp circular genome with 3047 predicted coding sequences (CDSs), 9 rRNA genes, 48 tRNA genes and a GC content of 64.8% (Supplementary Table S2; Figure 1). Of the 3047 CDSs, 79.65% were classified by Clusters of Orthologous Groups (COGs), and 681 (22.34%) were hypothetical proteins. It has been suggested that higher GC content may contribute towards reduced radiation susceptibility in various organisms, possibly through the formation of G-quadruplexes (Kumari et al., 2019; Kumari and Raghavan, 2021). In addition, 141 tandem repeats were predicted, accounting for 0.7580% of the total genome. There were 106 minisatellite and 6 microsatellite sequences, accounting for 0.170% and 0.0062% of the Ant6 genome, respectively (Supplementary Table S3). However, no CRISPR arrays were identified in the Ant6 genome. The phylogenetic tree revealed that the strain Ant6 formed a clade with *D. alpinitundrae* LMG 24283 (source: Alpine soil), *D. psychrotolerans* S14-83 **(**source: Antarctica soil), *D. detaillensis* H1 (source: Antarctica soil) and *D. irradiatisoli* 17bor-2 **(**source: gamma ray-irradiated soil). The present study showed that *D. alpinitundrae* LMG 24283 is the most closest relative of Ant6, which is consistent with previous reports based on 16s rRNA (Supplementary Figure S1) (Choi et al., 2016). The dDDH and OrthoANI values among the Ant6 and other related species were ranged between 14.30%–41.7% and 72.48–85.84%, respectively (Supplementary Table S4). These results further indicated that Ant6 represented a novel species under the genus *Deinococcus* (Choi et al., 2016).

### Comparative genome analysis

**FIGURE 2:**
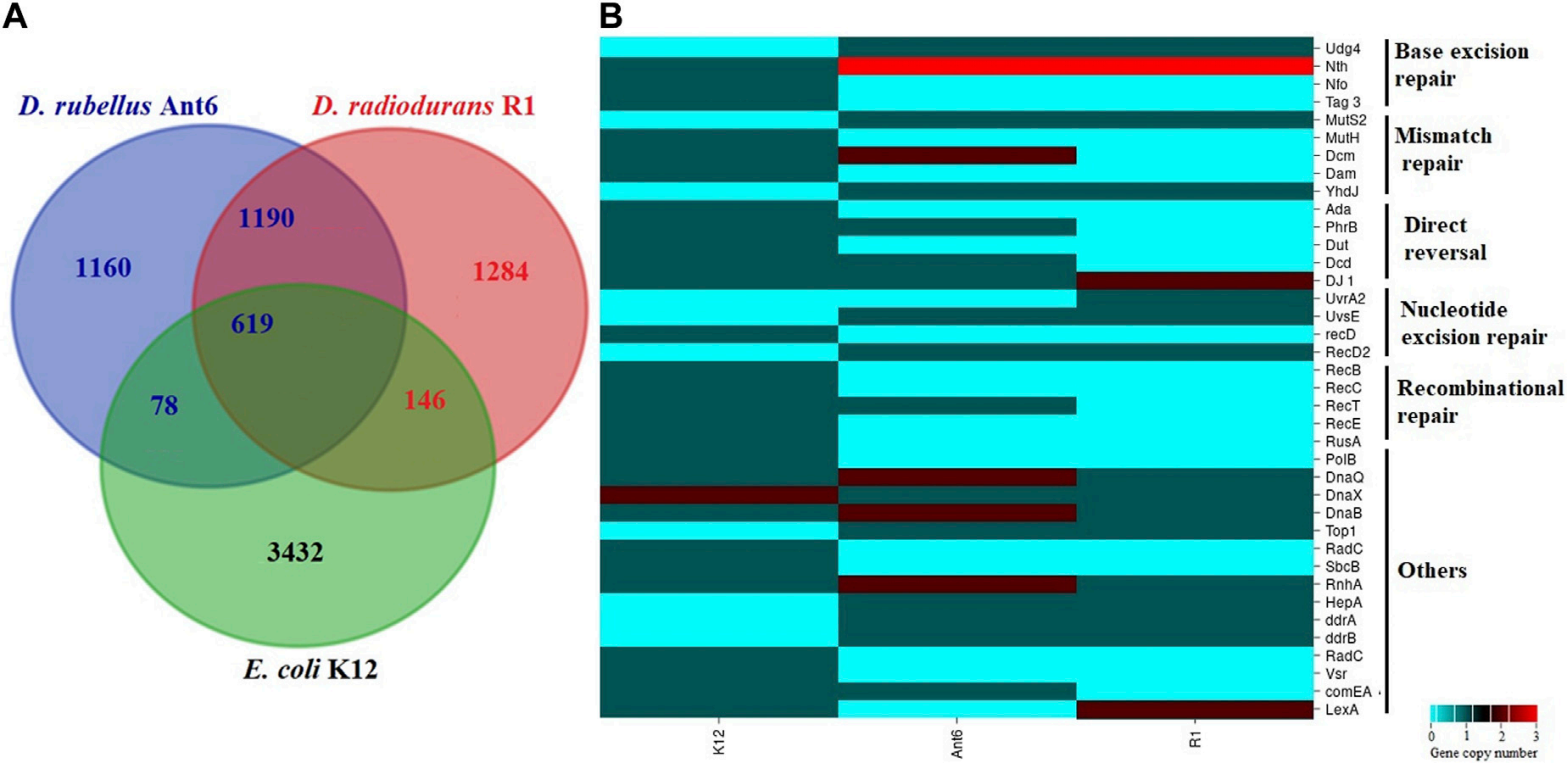
Comparative genome analysis of *Deinococcus rubellus* Ant6 with *Deinococcus radiodurans* R1*,* and *Escherichia coli* K12: **(A)** Comparison of Orthologous genes; **(B)** Main differences in DNA repair proteins.

To identify the functional gene categories associated with stress adaptation we compared the genome of *D. rubellus* Ant6 with *D. radiodurans* R1 and *E. coli* K12 (Supplementary Figure S2). Notably, the percentage of CDS associated with amino acid transport and metabolism, lipid transport and metabolism, secondary metabolite biosynthesis, defence metabolism, and signal transduction mechanisms were higher in the two *Deinococcus* species when compared to *E. coli*. In addition, Ant6 contains a higher percentage of genes associated with replication, recombination and repair, and cell motility whereas fewer genes for inorganic ion transport and metabolism, transcription, nucleotide transport and metabolism (Supplementary Figure S2). Putative orthologous genes among *D. rubellus* Ant6, *D*. *radiodurans* R1 and *E. coli* K12 were determined by reciprocal best-hits with a minimum of 30% identity and classified using Eggnog Mapper (Huerta-Cepas et al., 2017). A total of 1190 and 697 of the predicted gene products of *D. rubellus* Ant6 are homologous (at least 30% identity) to proteins from *D. radiodurans* R1 and *E. coli* K12, respectively whereas 619 *D. rubellus* Ant6 proteins are homologous to proteins from both *D. radiodurans* R1 and *E. coli* K12 (Figure 2A). This reveals a strong homologous relationship between the *D. rubellus* Ant6 and *D. radioduran*s R1 as compared to the radiosensitive strain *E*. *coli* K12. A total of 1160 *D. rubellus* Ant6 unique proteins were identified for which no homolog was detected in *D. radiodurans* R1 or *E. coli* K12. Most of these belong to hypothetical or mobile element proteins that probably play a key role in the characteristics of *D. rubellus* Ant6. Moreover, 1190 *D. rubellus* Ant6 proteins are homologous to proteins from *D. radiodurans* R1 but nonhomologous to *E. coli* K12 and these proteins could be the candidates for the phenotypic difference between radiation-resistant and radiation-sensitive bacteria.

### DNA repair and associated systems

Analysis of the Ant6 genome reveals the presence of a large number of DNA repair proteins participating in “classical” prokaryotic DNA repair machinery, but several interesting specific *D. rubellus* Ant6 features were also observed (Supplementary Table S5; Figure 2B). The base excision repair (BER) system of Ant6 encodes both monofunctional (*alkA, mutY, mug,* and *ung*) and bifunctional (*mutM* and *nth*) DNA glycosylase and AP endonuclease (*nfi* and *xthA*). Compared to *E. coli* K12, both *D. rubellus* Ant6 and *D. radiodurans* R1 strain encodes more than one variant of EndoIII proteins. The mismatch repair (MMR) system of Ant6 has a nearly identical proteome content with R1 and K12. However, MutS2, a paralogue of MutS was identified only in Ant6 and R1 but absent in the radiosensitive strain K12. Similarly, MutH, a key enzyme in the MMR pathway is missing in both Ant6 and R1 but present in K12. This indicates that the strand recognition system of *Deinococcus* is different from that of *E. coli*. A protein essential for repairing very short patch mismatches, specifically the Dcm methylase, was absent in *D. radiodurans*, showing the difference between the MMR systems of Ant6 and R1. Notably, Ant6 possesses deoxyribodipyrimidine photolyase (PhrB) involving direct reversal (DR) process are absent in R1. However, a previous study by Choi et al. (2016) showed that Ant6 is more susceptible to UV stress than R1. One possible explanation of this phenomenon, as suggested by the study on *Prochlorococcus* MED4, is that the presence of PhrB photolyase might not be the sole determinant of UV susceptibility and that other factors such as the upregulation of certain operons could play a role in UV susceptibility (Osburne et al., 2010). Additionally, the study on *Shewanella oneidensis* MR-1 highlights the role of prophages and oxidative damage in UV susceptibility (Qiu et al., 2005). Furthermore, the dual character of certain photolyases, as seen in *Rhodobacter sphaeroides*, suggests that the mere presence of a photolyase might not directly correlate with UV resistance (von Zadow et al., 2016). Similarly, the protein Deoxycytidine triphosphate deaminase (Dcd) that participates in the production of dTTP for DNA replication and damage repair is present in *D. rubellus* but absent in *D. radiodurans.* The NER system of Ant6 is similar to R1 and K12 involving uvrABC, uvrD, and transcription-repair coupling factor (superfamily II helicase) (mfd). Additionally, Ant6 and R1 encode a homolog of the endonuclease UvsE, a well-known repair protein that participates in UV-induced DNA damage and enhances radiation resistance in bacterial cells. UvrA2, a second UvrA that may have a minor role in UV resistance in R1, is absent in both Ant6 and K12 (Tanaka et al., 2005). The Ant6 strain encodes a set of essential genes for homologous recombination (HR), including the most homolog to known *E. coli*. However, *recB* and *recC* proteins are absent in the R1 and Ant6 genomes and members under the genus *Deinococcus* use RecFOR pathway for processing the double stranded DNA ends (Bentchikou et al., 2010).

Two major pathways have been identified for DSB repair in *D. radiodurans*: the RecA-independent single-strand annealing (SSA) pathway and the RecA-dependent extended synthesis-dependent strand annealing (ESDSA) pathway. During SSA repair, the DdrB protein is involved in the DNA annealing process, whereas DdrA prevents DNA degradation by the nucleases. This process also reduces the number of small DNA fragments and converts them into larger fragments, thereby creating a suitable substrate for a more accurate and efficient ESDSA repair pathway (Harris et al., 2004; Xu et al., 2010). Another study demonstrated upregulation of DNA repair proteins, including DdrA and DdrB following post-gamma irradiation and suggested that these proteins play a significant role in the organism’s DNA repair mechanism, particularly in protecting single-strand DNA fragments, which is a crucial line of defense of *D. radiodurans* (Basu and Apte, 2012). In the present study the proteins ddrA and ddrB were detected in Ant6 and R1 but not in K12. In ESDSA pathway, fragments with overlapping homologs are used to generate complementary single stranded fragment which anneals to form long linear intermediates and undergoes crossover to generate intact genome. Protein homologues associated with ESDSA such as RecA, UvrD, Pol I, PolII and RecJ are found to be conserve in the Ant6 genome (Zahradka et al., 2006). Unlike R1, the Ant6 genome contains error-prone DNA translesion polymerases such as PolB and DinP protein indicating the possible presence of an error-prone lesion bypass system.

Members under the genus *Deinococcus* were predicted to have several unique regulatory protein associated with radiation and oxidative stress response (Lim et al., 2019). Most bacteria use the RecA/Lexa-regulated SOS response to induce DNA repair genes to adapt to harsh environments. Under conditions without DNA damage stress, the SOS regulon genes are repressed by LexA. In contrast, when DNA is damaged, the coprotease activity RecA is activated, which inactivates the LexA repressor, leading to the expression of previously suppressed genes and initiation of DNA damage recovery (Erill et al., 2007). However, unlike in most bacteria, *D. radiodurans* lacks typical LexA/RecA mediated DNA damage response (SOS response) and cell cycle regulation. The LexA paralogs (DRA0344 and DRA0074) have been identified in *D. radiodurans* but none of them are induced nor implicated in RecA induction following exposure of ionizing radiation (Narumi et al., 2001; Sheng et al., 2004; Satoh et al., 2006). However, no lexA protein were identified in the genome of Ant6. It has been suggested that these bacteria may use alternative mechanisms to complement the absence of SOS repair mechanisms and cell cycle regulation. For example, in *D. radiodurans*, the induction of RecA and other repair genes occurs through an SOS independent mechanism and that this unique response system is regulated by two proteins DdrO and PprI (Ludanyi et al., 2014; Wang et al., 2015). The IrrE/DdrO system controls expressions of genes containing the RDRM (radiation-desiccation response motif) in the promoter region which is the binding sites of the transcriptional repressor protein DdrO. Once exposed to radiation or other oxidative stress condition, the IrrE peptidase cleaves which inactivates DdrO, leads to the expression of RDR genes (Makarova et al., 2007; Eugénie et al., 2021). The presence of the regulatory genes DdrO and PprI indicates a similar stress response system in Ant6.

The present study identifies nearly all the “Fts” proteins and the “Min” system like *E. coli* in both Ant6 and R1 genome (Supplementary Table S6), however, the regulatory mechanism associated with cell division in *Deinococcus* has not yet been fully elucidated. Mishra et al. (2022) reported that FtsK plays an important role in the genome separation, and septum formation and cell division in *D. radiodurans*. This protein was also identified in the Ant6 and K12 genome (Supplementary Table S6). In addition, the *Deinococcus* lacks important homologues to the known cell division regulatory proteins. For example, *SulA* **,** the SOS responsive protein attenuate the function of FtsZ under DNA damage stress and thus inhibiting cell division under DNA damage was absent in the genome of Ant6 and R1 (Chen et al., 2012). *Deinococcus* have also been reported to stop cell division in response to radiation stress; however, the exact mechanism of cell division arrest is not yet fully understood. Recent studies suggested that the phosphorylation of key cell division proteins such as FtsZ or DivIVA by the Serine/Threonine protein kinases (STPKs) (e.g., RqkA) could result in cell cycle arrest under radiation stress in *D. radiodurans* (Chaudhary et al., 2023a; Chaudhary et al., 2023b). Importantly, both the *Deinococcus* strain (Ant6 and R1) contains large number of STPK but these proteins were not detected in the radioresistance strain K12 (Supplementary Table S7). The RqkA protein (DR_2518) contains a PQQ-binding motifs and have been suggested to play an important role in the radiation resistance via DNA double-strand break (DSB) repair (Rajpurohit and Misra, 2010). These proteins were also present in Ant6, however, their functional role in cell cycle arrest and radiation resistance remains to be investigated. Overall, these evidences indicate the presence of unique DNA repair repertoires in Ant6 and R1 that contribute to survival under conditions of oxidative DNA damage.

### Reactive oxygen species detoxification

Analysis of the Ant6 genome reveals the presence of multiple enzymatic (superoxide dismutase, catalase, peroxiredoxin, thioredoxin, and glutaredoxin) and nonenzymatic antioxidant (Carotenoid, Bacillithiol, Manganese, and peptide) and pyrroloquinoline-quinone (PQQ) coenzymes that may play important roles in radiation protection (Misra et al., 2004; Slade and Radman, 2011) (Supplementary Table S8). The Strain Ant6 genome was predicted to encode a single gene for monofunctional heme catalases (UWX63958), whilst katA and katG were not detected. Superoxide dismutase was also encoded by a single gene ((Mn-containing Superoxide dismutases *sod*A (UWX65462)) in Ant6. Both superoxide dismutase and catalase have been reported to be associated with radiation resistance, and their activity is significantly higher in *Deinococcus* than in *E. coli* (Qi et al., 2020). The genome of Ant6 was predicted to encode bacterioferritin co-migratory protein and an atypical type of AhpC and alkyl hydroperoxidase D-like protein (YciW). These Peroxiredoxins (Prxs) proteins are known to catalyze the reduction of H_2_O_2_ or organic hydroperoxides (Meyer et al., 2009). Ant6 also encodes three OsmC (osmotically inducible protein C) family proteins: Ohr (UWX63319), OsmC (UWX63893), and YhfA (UWX63295). These OsmC proteins are commonly present in all *Deinococcus* species except for *D. proteolyticus* and are suggested to play a more important role in oxidative stress response than Ohr (Lim et al., 2019). The predicted Thioredoxins (Trxs) and Trx-related proteins in Ant6 were TrxA, TrxB, MsrA, MsrB, and HSP33. These proteins have disulfide reductase activity and protect cells against oxidative stress (Lu and Holmgren, 2014). A DsbA-FrnE protein (UWX63874) belonging to novel cytoplasmic oxidoreductases was identified in Ant6. Homologs of this protein (DR_0659) have shown upregulated expression when exposed to lethal doses of cadmium (Cd) in *D. radiodurans* (Joe et al., 2011). It has also been suggested that this protein plays an important role in bacterial resistance to Cd and other oxidative stress-generating substances (Khairnar et al., 2013). Moreover, the presence of nonenzymatic antioxidant carotenoid and bacillithiol in Ant6 could help the cell in ROS scavenging as well as restore the redox balance of proteins or lipids (Gaballa et al., 2010). Ant6 was predicted to encode ClpP, a well-known protein previously reported to play an important role in microbial adaptation to oxidative stress (Michel et al., 2006). Previous studies have shown that *D. radiodurans* is susceptible to radiation-induced DSBs, like other species, but its proteome is more tolerant to ROS-induced oxidative damage than radiation-sensitive species, suggesting that the proteome, rather that the genome, is the primary target responsible for *Deinococcus*’s survivability (Slade and Radman, 2011). The presence of a highly efficient antioxidant defence system in Ant6 genome may contribute to the survival in harsh environments. However, it is necessary to conduct functional analysis to reveal the actual role of these proteins in phenotypic characteristics of Ant6.

### Unique domain containing proteins

Various DNA repair proteins of *Deinococcus* have been reported to possess unique domain architectures that are structurally different from their homologues in other bacteria (Makarova et al., 2001). For example, most of the known RecQ proteins have only one HRDC domain but *D. radiodurans* R1 RecQ has three HRDC domains at its C-terminal region, and studies have shown that all three are associated with the high-affinity DNA binding and DNA unwinding process (Killoran and Keck, 2006). Interestingly *D. rubellus* Ant6 RecQ has only two HRDC domains indicating structural variation of RecQ proteins among *Deinococcus* (Supplementary Table S9). It has been reported that *D. radiodurans* recQ mutant is very sensitive to UV, mitomycin C (MMC), and H_2_O_2_. However, studies have reported that RecQ has little or no role in the IR resistance of *D. radiodurans* (Huang et al., 2007; Bentchikou et al., 2010). In addition, the *Deinococcus* proteins DR2444 (R1) and UWX64634 (Ant6) contain an HRDC domain and are not associated with a helicase or nuclease. Compared to *E. coli*, the RecD helicase in Ant6 and R1 has a long N terminal region and is termed RecD2. This protein is typically found in bacterial systems lacking the RecBCD complex which plays a major role in the repair of DNA double-strand breaks (Cox and Battista, 2005; Purkait et al., 2022). Moreover, *D. radiodurans* recD mutants are sensitive to IR, UV, and H_2_O_2_ stress. Another protein DRB0098 has been reported to possess an unusual domain structure containing a phosphatase domain of the HD superfamily and a polynucleotide kinase domain and suggested to play a role in the DNA repair process (Makarova et al., 2001). Several members of the *Deinococcus* extended protein family have been associated with proteins with unusual domain architectures. For example, many proteins of *D. radiodurans* contain endonuclease domain fused with additional domains such as RAD25-like helicase domain (DRA0131), SAD domain (DR1533), or a family of TerDEXZ/CABP family (DRA0057). The RAD25-like helicase domain is known to be involved in nucleotide excision repair of UV-damaged DNA in eukaryotes whereas the TerDEXZ/CABP family is suggested to be involved in stress response (Guzder et al., 1994; Makarova et al., 2001). However, these three unique proteins were not detected in the genome of Ant6 (Supplementary Table S9).

### Expanded protein families

Comparative analysis revealed the expansion of various families of hydrolases in the Ant6 genome as compared to the *E. coli* K12. The largest protein families expanded in Ant6 include signal transduction proteins, transcriptional regulators, several families of acetyltransferases, Nudix phosphohydrolase, Subtilisin-like protease, lipase-like (alpha/beta) hydrolases, calcineurine-like phosphoesterases, a stress response protein DinB/YfiT family, etc (Supplementary Figure S3). Most of these expanded protein families are proposed to be involved in cell cleaning functions, including the degradation of nucleic acids, proteins, or harmful products under stress conditions, and this feature may also help to enrich small intracellular molecules such as Mn, nucleosides, inorganic phosphates, and peptides, leading to the formation of Mn^2+^ metabolite complexes necessary to protect proteins from oxidative damage (Makarova et al., 2001; Daly et al., 2010).

## Conclusion

This is the first report of the whole-genome sequence of *D. rubellus* Ant6 isolated from the muscle of an Antarctic fish and highlights its survival mechanism under harsh environmental conditions. The genomic information obtained from the study can serve as a valuable resource to produce biotechnologically important biomolecules.

## Data Availability

The datasets presented in this study can be found in online repositories. The names of the repository/repositories and accession number(s) can be found below: https://www.ncbi.nlm.nih.gov/nuccore/CP104213.
