# Supplementary material for: Complete genome sequence of Deinococcus rubellus Ant6 isolated from the fish muscle in the Antarctic Ocean
Source: Front Bioeng Biotechnol. 2023 Oct 16;11:1257705. doi: 10.3389/fbioe.2023.1257705 (PMC10614293; doi:10.3389/fbioe.2023.1257705)
Supplement: Supplementary file 1 [file DataSheet1.docx]

Supplementary Material

**Complete genome sequence of *Deinococcus rubellus* Ant6 isolated from the fish muscle in the Antarctic Ocean**

Surajit De Mandal^1^, Sathiyaraj Srinivasan^2^* and Junhyun Jeon^1,3^*

^1^Department of Biotechnology, Yeungnam University, Gyeongsan, Gyeongbuk, 38541, Korea

^2^Department of Bio & Environmental Technology, College of Natural Science, Seoul Women’s University, Seoul 01797, Korea

^3^Plant Immunity Research Center, Seoul National University, Seoul, 08826, Korea

*** Correspondence:**Junhyun Jeon, Phone: +82-2-810-3030; Fax: +82-53-810-4769; E-mail: [jjeon@yu.ac.kr](mailto:jjeon@ynu.ac.kr)

Sathiyaraj Srinivasan, Phone: +82 -2-970-5670; Fax: +82-2-970-5974; Email: [drsrini@swu.ac.kr](mailto:drsrini@swu.ac.kr)

**Supplementary Figure 1.** Phylogenetic tree analysis of *D. rubellus* Ant6 and related species.


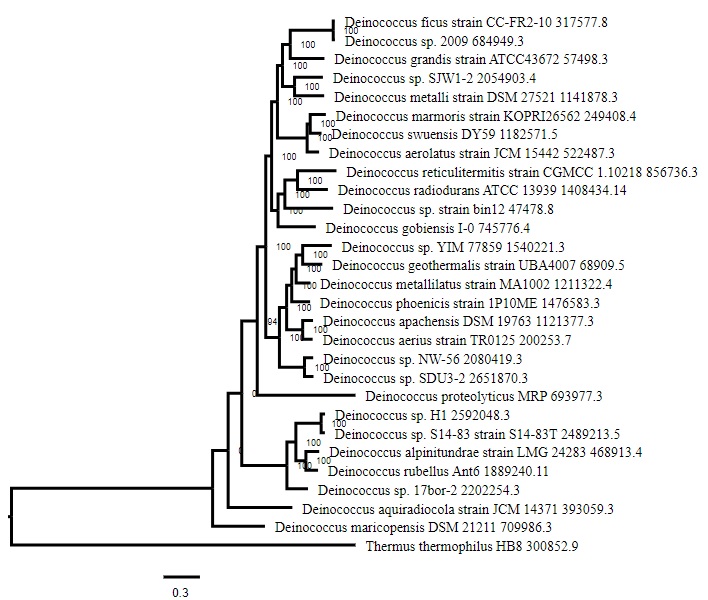


**Supplementary Figure 2.** Comparison of COG functional categories among *D. rubellus* Ant6, *D. radiodurans* R1*,* and *E. coli* K12.


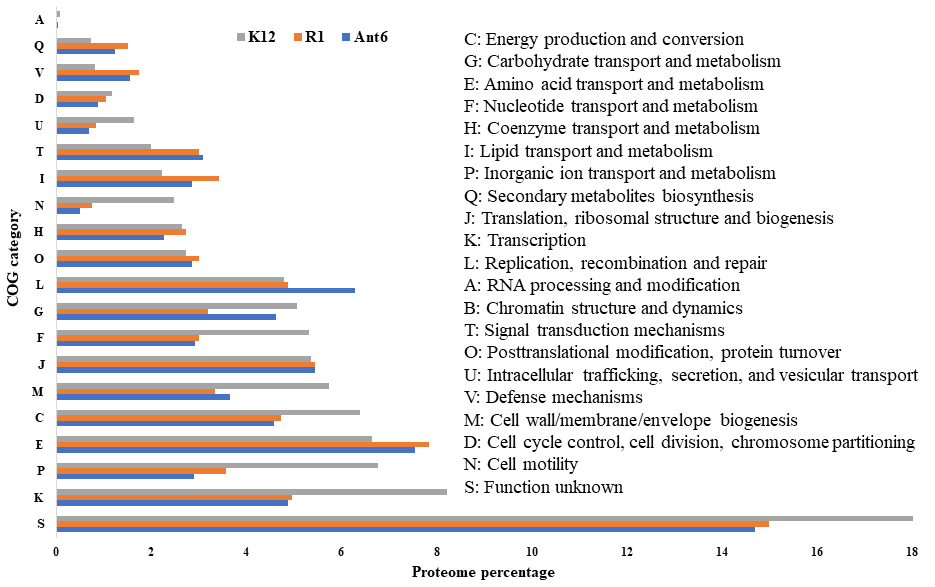


**Supplementary Figure 3.** Comparison of extended protein families in *D. rubellus* Ant6 with *D. radioduran* R1 and *E. coli* K12.


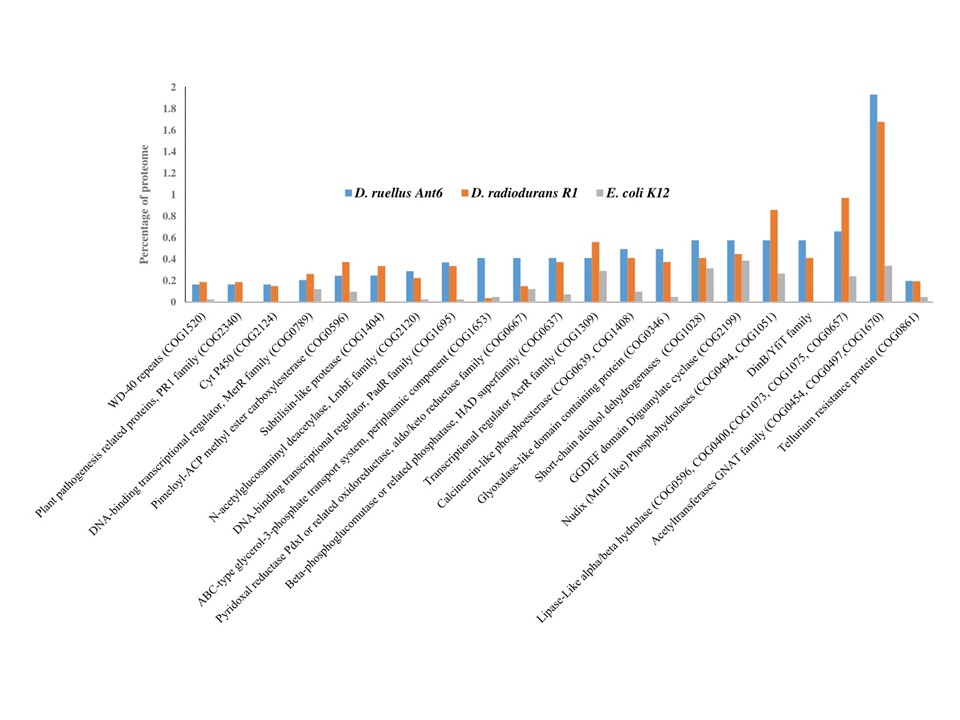


**Supplementary table 1:** D10 values and the optimal growth temperature of *Deinococcus* *rubellus* Ant6, *Deinococcus radioduran* R1and *Escherichia coli* K12.

| **Strain name** | **D10 value / Optimal growth temperature** | **References** |
| --- | --- | --- |
| *Deinococcus* *rubellus* Ant6 | 4 kGy (20°C) | Choi et al., 2016 |
| *Deinococcus radioduran* R1 | 15 kGy (20°C) | Makarova et al., 2007 |
| *Escherichia coli* K12 | 0.7 kGy (37°C) | Daly et al., 2004 |

**Supplementary table 2:** General features of the *Deinococcus rubellus* Ant6 genome.

| **Attribute** | **Values** |
| --- | --- |
| Genome size (bp) | 3041811 |
| GC content (%) | 64.8 |
| rRNA genes | 9 |
| tRNA genes | 48 |
| ncRNA | 2 |
| tmRNA | 1 |
| CDS | 3047 |
| Hypothetical proteins | 681 |
| Proteins with functional assignments | 2366 |
| Regulatory | 4 |

**Supplementary table 3:** Statistics analysis of repeat sequence in *Deinococcus rubellus* Ant6

| Type | Number | Repeat Size (bp) | Total Length (bp) | Genome (%) |
| --- | --- | --- | --- | --- |
| TRF | 141 | 5-270 | 8470.3 | 0.879 |
| Minisatellite DNA | 106 | 15-65 | 5183.5 | 0.170 |
| Microsatellite DNA | 6 | 7-10 | 207 | 0.006 |

**Supplementary table 4:** **Average nucleotide identity (ANI) and digital DNA–DNA hybridization (dDDH) values between strain Ant6 and its closely related strains**

| **Strain name** | **ANIb** | **dDDH** |
| --- | --- | --- |
| *D. alpinitundrae* LMG 24283 | 85.84 | 41.7 |
| *D. psychrotolerans* S14-83 | 79.51 | 23.7 |
| *D. detaillensis* H1 | 79.48 | 25.4 |
| *D. irradiatisoli* 17bor-2 | 80.23 | 37.1 |
| *D. radiodurans* R1 | 72.48 | 14.3 |

**Supplementary Table 5.** DNA repair proteins in *Deinococcus* *rubellus* Ant6, *Deinococcus radioduran* R1and *Escherichia coli* K12

| **Pathway** | **Protein Description** | ***D. radiodurans* R1** | ***D. rubellus* Ant6** | ***E. coli* K12** | **COG number** |
| --- | --- | --- | --- | --- | --- |
| **DNA Repair proteins for Base excision repair** | | | | | |
| AlkA | DNA-3-methyladenine glycosylase 2 | AAF12123 | UWX64472 | AAC75129 | COG0122 |
| Mpg | DNA-3-methyladenine glycosylase | AAF11623 | UWX63019 | - | COG2094 |
| Ung | Uracil DNA glycosylase | AAF10269 | UWX64851 | AAC75633 | COG0692 |
| Udg4 | Uracil-DNA glycosylase | AAF11304 | UWX63274 | - | COG1573 |
| MutY | Adenine-specific DNA glycosylase, acts on AG and A-oxoG pairs | AAF11831 | UWX64632 | AAC75998 | COG1194 |
| Mug | G:T/U-mismatch repair DNA glycosylase | AAF10293 | UWX62956 | AAC76104 | COG3663 |
| MutY | Adenine-specific DNA glycosylase, acts on AG and A-oxoG pairs | AAF11831 | UWX64632 | AAC75998 | COG1194 |
| MutM | Formamidopyrimidine-DNA glycosylase | AAF10070 | UWX62810 | AAC76659 | COG0266 |
| Nth | Endonuclease III | AAF09870  AAF10505  AAF11977 | UWX62810  UWX62710  UWX63293 | AAC74705 | COG0177 |
| Nfi | Deoxyinosine 3'endonuclease (endonuclease V) | AAF11706 | UWX63256 | AAC76972 | COG1515 |
| XthA | Exonuclease III | AAF09936 | UWX63989 | AAC74819 | COG0708 |
| **DNA Repair proteins for Mismatch Repair** | | | | | |
| MutL | DNA mismatch repair ATPase | AAF11253 | UWX65467 | AAC77127 | COG0323 |
| MutS | DNA mismatch repair ATPase | UDL00179 | UWX65466 | AAC75775 | COG0249 |
| MutS2 | dsDNA-specific endonuclease/ATPase | AAF11527 | UWX63997 | - | COG1193 |
| YcjD | endonuclease domain-containing protein (DUF559) | AAF09807 | UWX64776 | AAC74371 | COG2852 |
| MutH | DNA mismatch repair protein MutH | - | - | AAC75870 | COG3066 |
| Dcm | Site-specific DNA-cytosine methylase | - | UWX64534 UWX62749 | AAC75027 | COG0270 |
| Dam | Site-specific DNA-adenine methylase | - | - | AAC76412 | COG0338 |
| **DNA Repair proteins for Direct reversal** | | | | | |
| Ada | Bifunctional transcriptional activator/DNA repair enzyme | - | - | AAC75273 | COG2169/ COG0350 |
| PhrB | Deoxyribodipyrimidine photolyase | - | UWX63182 | AAC73802 | COG0415 |
| Dut | dUTPase | - | - | AAC76664 | COG0756 |
| Dcd | Deoxycytidine triphosphate deaminase | - | UWX65281 | AAC75126 | COG0717 |
| HAM1/YggV | xantosine triphosphate pyrophosphatase, prevents 6-N-hydroxylaminopurin mutagenesis | AAF09767 | UWX62958. | AAC75991 | COG0127 |
| DJ-1/PfpI family | methylglyoxal and glyoxal deglycase | AAF10068  AAF10772 | UWX63636 | AAC76187 | COG0693 |
| **DNA Repair proteins for Nucleotide Excision Repair (NER)** | | | | | |
| UvrA | Excinuclease UvrABC, ATPase subunit | AAF11324 | UWX65492 | AAC77028 | COG0178 |
| UvrA2 | Excinuclease UvrABC, ATPase subunit | AAF12187 | - | - | COG0178 |
| UvrB | Excinuclease UvrABC, helicase subunit | AAF11823 | UWX64535 | AAC73866 | COG0556 |
| UvrC | Excinuclease UvrABC, nuclease subunit | AAF10924 | UWX63091 | AAC74980 | COG0322 |
| UvrD | Superfamily I DNA or RNA helicase | AAF11329 | UWX65508 | AAC76816 | COG0210 |
| Mfd | Transcription-repair coupling factor (superfamily II helicase) | AAF11095 | UWX63026 | AAC74198 | COG1197 |
| UvsE | UV DNA damage repair endonuclease | AAF11370 | UWX63534 | - | COG4294 |
| Atl1 | Alkylated DNA nucleotide flippase Atl1, Ada-like DNA-binding domain | AAF10005 | UWX64856 | AAC73557 | COG3695 |
| **DNA repair genes for recombinational repair (RER)** | | | | | |
| RecA | Protein RecA; Recombinase A | AAF11887 | UWX63662 | AAC75741 | COG0468 |
| RecF | Recombinational DNA repair ATPase RecF | AAF10663 | UWX62841 | AAC76723 | COG1195 |
| RecO | DNA repair protein RecO; Recombination protein O | AAF10403 | UWX63405 | AAC75618 | COG1381 |
| RecR | Recombinational DNA repair protein RecR | AAF09785 | UWX64960 | AAC73574 | COG0353 |
| RecJ | Single-stranded DNA-specific exonuclease | AAF10698 | UWX65594 | AAC75930 | COG0608 |
| RecN | DNA repair ATPase RecN | AAF11043 | UWX63653 | AAT48145 | COG0497 |
| RecQ | Superfamily II DNA helicase RecQ (including HRDC domains) | AAF10859 | UWX63339 | AAT48221 | COG0514 |
| RecD | ATP-dependent RecD-like DNA helicase | AAF11453 | UWX63593 | AAC75858 | COG0507 |
| SbcC | DNA repair exonuclease SbcCD, ATPase subunit | AAF11474 | UWX63972 | AAC73500 | COG0419 |
| SbcD | DNA repair exonuclease SbcCD, nuclease subunit | AAF11473 | UWX63971 | AAC73501 | COG0420 |
| RuvA | Holliday junction resolvasome RuvABC, DNA-binding subunit | AAF10840 | UWX65034 | AAC74931 | COG0632 |
| RuvB | Holliday junction resolvasome RuvABC, DNA helicase subunit | AAF10176 | UWX65099 | AAC74930 | COG2255 |
| RuvC | Holliday junction resolvasome RuvABC, endonuclease subunit | AAF10018 | UWX64413 | AAC74933. | COG0817 |
| RecG | RecG-like helicase | AAF11469 | UWX63476 | AAC76676 | COG120 |
| RecB | ATP-dependent exoDNAse (exonuclease V) beta subunit | - | - | AAC75859 | COG1074 |
| RecC | Exonuclease V gamma subunit | - | - | AAC75861 | COG1330 |
| RecT | Recombinational DNA repair protein RecT | - | - | AAC74431 | COG3723 |
| RecX | SOS response regulatory protein OraA/RecX, interacts with RecA | AAF10882 | UWX64916 | AAC75740 | COG2137 |
| RadA | DNA repair protein RadA | AAF10678 | UWX64974 | AAC77342 | COG1066 |
| RusA | Holliday junction resolvase RusA | -- | - | AAC73651 | COG4570 |
| **Other DNA repair related genes** | | | | | |
| PolA | DNA polymerase I - 3'-5' exonuclease and polymerase domains | AAF11264 | UWX62713 | AAC76861 | COG0749 |
| PolB | DNA polymerase elongation subunit (family B) | - | - | AAC73171 | COG0417 |
| DnaA | Chromosomal replication initiator protein | AAF09596 | UWX64433 | AAC76725 | COG0593 |
| DnaB | Replicative DNA helicase | AAF10128 | UWX65429  UWX62752 | AAC77022 | COG0305 |
| DnaE | DNA polymerase III, alpha subunit | AAF10085 | UWX64532 | AAC73295 | COG0587 |
| DnaG | DNA primase | AAF10180 | UWX63747 | AAC76102 | COG0358 |
| DnaQ | DNA polymerase III, epsilon subunit or related 3'-5' exonuclease | AAF10431 | UWX63596  UWX65680 | AAC74914 | COG0847 |
|  |  |  |  |  |  |
|  |  |  |  |  |  |
| DnaN | DNA polymerase III sliding clamp (beta) subunit, PCNA homolog | AAF09595 | UWX64434 | AAC76724 | COG0592 |
| DnaX | DNA polymerase III, gamma/tau subunits | AAF11953 | UWX65136 | AAC73572  AYC08179 | COG2812 |
| Ssb | Single-stranded DNA-binding protein | AAF09692 | UWX64566 | - | COG0629 |
| GyrA | DNA gyrase/topoisomerase IV, subunit A | AAF11467 | UWX62838 | AAC75291 | COG0188 |
| GyrB | DNA gyrase/topoisomerase IV, subunit B | AAF10481 | UWX65500 | AAT48201 | COG0187 |
| Top1 | DNA topoisomerase IB | AAF10270 | UWX64852 | - | COG3569 |
| TopA | DNA topoisomerase IA/Uncharacterized C-terminal domain of TopIA | AAF10943 | UWX65405 | AAC74356 | COG0550/ COG1754 |
| RarA | Replication-associated recombination protein RarA | AAF11452 | UWX63495 | AAC73978 | COG2256 |
| RadC | DNA repair protein containing a helix-hairpin-helix DNA-binding motif | - | - | AAC76662 | COG2003 |
| Obg | GTPase involved in cell partioning and DNA repair | AAF09676 | UWX64349 | AAC76215 | COG0536 |
| MutK | Uncharacterized membrane protein (maybe involved in DNA repair) | AAF10869 | UWX64829 | AAC75024 | COG2354 |
| XseA | Exonuclease VII, large subunit | AAF09773 | UWX64519 | AAC75562 | COG1570 |
| XseB | Exonuclease VII small subunit | AAF12134 | UWX64346 | AAF12134 | COG1722 |
| SbcB | Exonuclease I | - | - | AAC75072 | COG2925 |
| YqgF | RNase H-fold protein, predicted Holliday junction resolvase | AAF12050 | UWX65663 | AAC75986 | COG0816 |
| RnhA | Ribonuclease HI | AAF10474 | UWX63812  UWX62984 | AAC73319 | COG0328 |
| RnhB | Ribonuclease HII | AAF11501 | UWX65531 | AAC73294 | COG0164 |
| HepA | Superfamily II DNA or RNA helicase, SNF2 family | AAF10830 | UWX65055 | - | COG0553 |
| PprI | ImmA/IrrE family metallo-endopeptidase | AAF09762 | UWX65598 | - | COG2856 |
| DdrO | transcriptional regulator, | AAF12112 | UWX63829 | - | COG1396 |
| DdrA | single-stranded DNA-binding protein | [AE000513](https://www.ncbi.nlm.nih.gov/nuccore/AE000513.1) | [UWX63081](https://www.ncbi.nlm.nih.gov/protein/UWX63081.1) | - | COG4712 |
| DdrB | single-stranded DNA-binding protein | [AE000513](https://www.ncbi.nlm.nih.gov/nuccore/AE000513.1) | [UWX64889](https://www.ncbi.nlm.nih.gov/protein/UWX64889.1) | - | - |
| DdrC | DNA damage response protein | ANC70345 | UWX63658 | - | - |
| DdrD | DNA damage response protein | AAF09913 | UWX64658 | - | - |
| DdrH | hypothetical protein | - | [UWX63836](https://www.ncbi.nlm.nih.gov/protein/UWX63836.1) | - | COG1670 |
| DdrI | CRP/Fnr family transcriptional regulator | - | [UWX62837](https://www.ncbi.nlm.nih.gov/protein/UWX62837.1) | - | COG0664 |
| DdrN | N-acetyltransferase GCN5 | - | [UWX64261](https://www.ncbi.nlm.nih.gov/protein/UWX64261.1) | - | - |
| DdrO | transcriptional repressor of the RDR regulon | - | [UWX63829](https://www.ncbi.nlm.nih.gov/protein/UWX63829.1) | - | COG1396 |
| DinP | DNA polymerase IV | - | UWX62806 | AAC73335 | COG0389 |

**Supplementary table 6 : Major** **cell division and genome segregation related proteins in *Deinococcus***

| **Pretein names** | **Protein descriptions** | ***D. radiodurans* R1** | ***D. rubellus* Ant6** | ***E. coli* K12** | **COG numbers** |
| --- | --- | --- | --- | --- | --- |
| DivIVA | DivIVA domain-containing protein | AAF10941.1 | UWX63387.1 | - | COG3599 |
| FtsZ | cell division protein | AAF10211.1 | UWX65306.1 | AAC73206.1 | COG0206 |
| FtsA | cell division protein FtsA | AAF10210.1 | UWX63901.1 | AAC73205.1 | COG0849 |
| ftsE | ftsE protein | AAF11110.1 | UWX63685.1 | AAC76487.1 | COG2177 |
| ftsK | cell division protein FtsK | AAF09980.1 | UWX63726.1 | AAC73976.1 | COG1674 |
| FtsW | FtsW/RodA/SpoVE family cell division protein, | AAF12039.1 | UWX65668.1 | AAC73200.1 | COG0772 |
| FtsQ | cell division related protein | AAF10209.1 | UWX63900.1 | AAC73204.1 | COG1589 |
| MinD | septum site-determining protein | AAF10331.1 | UWX63168.1 | AAC74259.1 | COG2894 |
| MinC | septum site-determining protein, putative | AAF11310.1 | UWX65473.1 | AAC74260.1 | COG0850 |
| ParA1 | chromosome partitioning ATPase | AAF09606.1 | UWX64555.1 | - | COG1192 |
| ParA2 | chromosome partitioning ATPase | AAF12301.1 | UWX63396.1 | - | COG1192 |
| ParA3 | chromosome partitioning ATPase | AAF12609.1 | - | - | COG1192 |
| ParA4 | chromosome partitioning ATPase | AAF12584.1 | - | - | COG1192 |
| ParB1 | chromosome partitioning ATPase | AAF09605.1 | UWX64556.1 | - | COG1475 |
| ParB2 | chromosome partitioning protein | AAF12300.1 |  | - | COG1475 |
| ParB3 | chromosome partitioning protein | AAF12610.1 | - | - | COG1475 |
| ParB4 | chromosome partitioning protein | AAF12599.1 |  |  | COG1475 |

**Supplementary table 7 : Kinase proteins predicted in *Deinococcus* *rubellus* Ant6, *Deinococcus radioduran* R1 and *Escherichia coli* K12**

| **Protein descriptions** | ***D. radiodurans* R1** | ***D. rubellus* Ant6** | ***E. coli* K12** |
| --- | --- | --- | --- |
| [LysW]-aminoadipate kinase | AAF10984.1 | UWX62724.1 | AAC76941.3 |
| 2-amino-4-hydroxy-6-hydroxymethyldihydropteridine pyrophosphokinase (EC 2.7.6.3) | AAF09758.1 | UWX65015.1 | AAC73253.1 |
| 6-phosphofructokinase (EC 2.7.1.11) | AAF10213.1 | UWX64031.1 | AAC76898.1 |
| Aspartokinase (EC 2.7.2.4) | AAF10936.1 | UWX63060.1 | AAC73113.1 |
| Aspartokinase (EC 2.7.2.4) | - | - | AAC76994.1 |
| Branched-chain acyl kinase | AAF10694.1 | UWX63525.1 | - |
| Copper sensory histidine kinase CusS | AAF10322.1 | UWX63624.1 | AAC73671.1 |
| Deoxyadenosine kinase (EC 2.7.1.76) @ Deoxyguanosine kinase (EC 2.7.1.113) | AAF09882.1 | UWX64278.1 | - |
| Deoxyadenosine kinase (EC 2.7.1.76) @ Deoxyguanosine kinase (EC 2.7.1.113) | AAF09883.1 | UWX64279.1 | - |
| Dihydroxyacetone kinase-like protein, phosphatase domain / Dihydroxyacetone kinase-like protein, kinase domain | AAF09965.1 | UWX65633.1 | - |
| FMN adenylyltransferase (EC 2.7.7.2) / Riboflavin kinase (EC 2.7.1.26) | AAF10583.1 | UWX65304.1 | AAC73136.1 |
| Fructokinase (EC 2.7.1.4) | AAF10301.1 | UWX63713.1 | - |
| Homoserine kinase (EC 2.7.1.39) | AAF09972.1 | - | - |
| Multidomain signal transduction protein including CheB-like methylesterase, CheR-like methyltransferase and BaeS-like histidine kinase | AAF10744.1 | UWX62828.1 | - |
| Pantothenate kinase type III, CoaX-like (EC 2.7.1.33) | AAF10040.1 | UWX65111.1 | - |
| Phosphoenolpyruvate carboxykinase [ATP] (EC 4.1.1.49) | AAF10554.1 | UWX63887.1 | AAC76428.1 |
| Phosphoglycerate kinase (EC 2.7.2.3) | AAF10913.1 | UWX63078.1 | AAC75963.1 |
| Phosphoglycerate kinase (EC 2.7.2.3) | AAF11081.1 | UWX64864.1 | - |
| Polyphosphate glucokinase (EC 2.7.1.63) | AAF10398.1 | UWX63804.1 | AAC74203.1 |
| putative periplasmic protein kinase ArgK and related GTPases of G3E family | AAF10633.1 | UWX65301.1 | AAC75955.1 |
| Serine/threonine protein kinase | AAF10786.1 | UWX64044.1 | - |
| Serine/threonine protein kinase | AAF10814.1 | UWX62877.1 | - |
| Ribose-phosphate pyrophosphokinase (EC 2.7.6.1) | AAF11025.1 | UWX65107.1 | AAC74291.1 |
| Sensor histidine kinase/response regulator | AAF10748.1 | UWX63296.1 | - |
| Serine/threonine protein kinase | AAF09648.1 | UWX63912.1 | - |
| Serine/threonine protein kinase | AAF09932.1 | UWX65016.1 | - |
| Serine/threonine protein kinase | AAF10786.1 | UWX64044.1 | - |
| Shikimate kinase I (EC 2.7.1.71) | AAF10352.1 | UWX65178.1 | AAC76415.2 |
| Thiamin pyrophosphokinase (EC 2.7.6.2) | AAF10131.1 | UWX65006.1 | - |
| Ribokinase (EC 2.7.1.15) | AAF11090.1 | UWX63993.1 | AAC74842.2 |
| Diacylglycerol kinase-related protein | AAF11121.1 | UWX63877.1 | - |
| putative periplasmic protein kinase ArgK and related GTPases of G3E family | AAF11302.1 | UWX65301.1 | - |
| Serine/threonine protein kinase-related protein | AAF11377.1 | - | - |
| Glutamate 5-kinase (EC 2.7.2.11) / RNA-binding C-terminal domain PUA | AAF11381.1 | UWX62873.1 | AAC73346.1 |
| Guanosine-3',5'-bis(diphosphate) 3'-pyrophosphohydrolase (EC 3.1.7.2) / GTP pyrophosphokinase (EC 2.7.6.5), (p)ppGpp synthetase II | AAF11392.1 | UWX63815.1 | AAC76674.1 |
| GDP/GTP pyrophosphokinase | - | - | AAC75826.1 |
| Serine/threonine protein kinase-related protein | AAF11404.1 | UWX63945.1 | - |
| Dephospho-CoA kinase (EC 2.7.1.24) | AAF11446.1 | UWX62913.1 | AAC73214.1 |
| Gluconokinase (EC 2.7.1.12) | AAF11464.1 | UWX63535.1 | AAC76462.2 |
| putative kinase | AAF11472.1 | - | - |
| Glycerol kinase (EC 2.7.1.30) | AAF11475.1 | UWX64404.1 | AAC76908.1 |
| Thymidine kinase (EC 2.7.1.21) | AAF11536.1 | UWX63407.1 | - |
| Adenylate kinase (EC 2.7.4.3) | AAF11666.1 | UWX64684.1 | AAC73576.1 |
| Guanylate kinase (EC 2.7.4.8) | AAF11836.1 | UWX64258.1 | AAC76672.1 |
| Cryptic sugar kinase Mak | AAF11841.1 | UWX64127.1 | AAC73497.2 |
| Hexokinase (EC 2.7.1.1) | - | - | AAC73497.2 |
| N-acetyl-D-glucosamine kinase (EC 2.7.1.59) | - | - | AAC74203.1 |
| Pseudouridine kinase (EC 2.7.1.83) | AAF11859.1 | UWX65101.1 | AAC75221.1 |
| N-acetylmannosamine kinase (EC 2.7.1.60) | - | UWX64363.1 | AAC76254.2 |
| Homoserine kinase (EC 2.7.1.39) | AAF11935.1 | UWX65441.1 | AAC73114.1 |
| sensory box sensor histidine kinase | AAF11962.1 | - | - |
| N-acetylglutamate kinase (EC 2.7.2.8) | AAF11985.1 | UWX64262.1 | AAC76941.3 |
| N-acetylglutamate kinase (EC 2.7.2.8) | AAF11985.1 | UWX64262.1 | AAC76941.3 |
| Nucleoside diphosphate kinase (EC 2.7.4.6) | AAF12041.1 | UWX63230.1 | AAC75571.1 |
| Serine/threonine protein kinase PrkC, regulator of stationary phase | AAF12057.1 | UWX65058.1 | - |
| Cytidylate kinase (EC 2.7.4.25) | AAF12083.1 | UWX64878.1 | AAC73996.1 |
| Ribokinase (EC 2.7.1.15) | AAF12093.1 | UWX64879.1 | - |
| Acetate kinase (EC 2.7.2.1) | AAF12139.1 | UWX63088.1 | AAC75356.1 |
| Acetate kinase (EC 2.7.2.1) @ Propionate kinase (EC 2.7.2.15) | - | - | AAC76150.2 |
| 4-diphosphocytidyl-2-C-methyl-D-erythritol kinase (EC 2.7.1.148) | AAF12142.1 | UWX63985.1 | AAC74292.1 |
| Pyruvate kinase (EC 2.7.1.40) | AAF12171.1 | UWX64436.1 | AAC74746.1 |
| Pyruvate kinase (EC 2.7.1.40) | - | - | AAC74924.1 |
| Phytochrome, two-component sensor histidine kinase (EC 2.7.3.-) | AAF12175.1 | UWX64999.1 | - |
| Pyridoxal kinase (EC 2.7.1.35) | AAF12189.1 | UWX64329.1 | AAC74708.1 |
| Hydroxymethylpyrimidine phosphate kinase ThiD (EC 2.7.4.7) | AAF12199.1 | - | AAC75164.1 |
| 6-deoxy-6-sulphofructose kinase | AAF12258.1 | - | AAD13445.2 |
| Ribokinase (EC 2.7.1.15) | - | - | AAC76775.1 |
| Ribokinase (EC 2.7.1.15) | AAF12258.1 | UWX64593.1 | AAC76775.1 |
| Phytochrome, two-component sensor histidine kinase (EC 2.7.3.-) | AAF12261.1 |  |  |
| Adenosylcobinamide kinase (EC 2.7.1.156) / Adenosylcobinamide-phosphate guanylyltransferase (EC 2.7.7.62) | AAF12282.1 | - | AAC75054.1 |
| Adenylylsulfate kinase (EC 2.7.1.25) | AAF12298.1 | UWX63857.1 | AAC75792.1 |
| protein kinase, putative | AAF12449.1 | - | - |
| Osmosensitive K+ channel histidine kinase KdpD | AAF12538.1 | - | - |
| Sensor histidine kinase/response regulator | AAF12583.1 | - | - |
| 1-phosphofructokinase (EC 2.7.1.56) | AAF12596.1 | - | AAC75229.1 |
| 6-phosphofructokinase class II (EC 2.7.1.11) | - | - | AAC74793.1 |
| 2-dehydro-3-deoxygalactonokinase (EC 2.7.1.58) | - | - | AAC76716.1 |
| 2-dehydro-3-deoxygluconokinase (EC 2.7.1.45) | - | - | AAC76551.2 |
| 3-oxo-tetronate kinase | - | - | AAC75779.1 |
| Anhydro-N-acetylmuramic acid kinase (EC 2.7.1.170) | - | - | AAC74712.1 |
| Aspartokinase (EC 2.7.2.4) / Homoserine dehydrogenase (EC 1.1.1.3) | - | - | AAC76922.1 |
| Autoinducer 2 (AI-2) kinase LsrK (EC 2.7.1.-) | - | - | AAC74584.1 |
| Carbamate kinase (EC 2.7.2.2) | - | - | AAC73623.1 |
| Carbamate kinase-like protein YahI | - | - | AAC73426.1 |
| Carbamate kinase-like protein YahI | - | - | AAC73426.1 |
| Carbamate kinase-like protein YqeA | - | - | AAC75912.1 |
| Copper sensory histidine kinase CpxA | - | - | AAC76893.1 |
| D-glycero-beta-D-manno-heptose 1-phosphate adenylyltransferase (EC 2.7.7.70) / D-glycero-beta-D-manno-heptose-7-phosphate kinase (EC 2.7.1.167) | - | - | AAC76088.1 |
| Diacylglycerol kinase (EC 2.7.1.107) | - | UWX65270.1 | AAC77012.1 |
| FIG001592: Phosphocarrier protein kinase/phosphorylase, nitrogen regulation associated | - | - | AAC75868.1 |
| Fumarate respiration sensor kinase protein DcuS | - | - | AAC77086.1 |
| Galactokinase (EC 2.7.1.6) | - | - | AAC73844.1 |
| Glucokinase (EC 2.7.1.2) | - | - | AAC75447.1 |
| Gluconokinase (EC 2.7.1.12) | - | - | AAC76462.2 |
| Gluconokinase (EC 2.7.1.12) | - | - | AAC77225.1 |
| Glycerate kinase (EC 2.7.1.31) | - | - | AAC73616.1 |
| Glycerate-2-kinase (EC 2.7.1.165) | - | - | AAC76158.2 |
| Hexose-phosphate uptake signal transduction histidine-protein kinase/phosphatase UhpB | - | - | AAC76691.2 |
| Hybrid sensory histidine kinase in two-component regulatory system with EvgA | - | - | AAC75429.1 |
| Hydroxyethylthiazole kinase (EC 2.7.1.50) | - | - | AAC75165.1 |
| Inosine-guanosine kinase (EC 2.7.1.73) | - | - | AAC73579.1 |
| Isocitrate dehydrogenase phosphatase (EC 2.7.11.5)/kinase (EC 3.1.3.-) | - | - | AAC76986.1 |
| L-fuculokinase (EC 2.7.1.51) | - | - | AAC75845.2 |
| Lipid kinase YegS | - | - | AAC75147.1 |
| Lipopolysaccharide core heptose(I) kinase RfaP | - | - | AAC76654.1 |
| Lipopolysaccharide core heptose(II) kinase RfaY | - | - | AAC76649.1 |
| L-xylulose/3-keto-L-gulonate kinase (EC 2.7.1.-) | - | - | AAC76604.1 |
| Multidomain signal transduction protein including CheB-like methylesterase, CheR-like methyltransferase and BaeS-like histidine kinase | - | UWX65329.1 | - |
| Multidomain signal transduction protein including CheB-like methylesterase, CheR-like methyltransferase and BaeS-like histidine kinase | - | UWX65330.1 | - |
| NAD kinase (EC 2.7.1.23) | - | - | AAC75664.1 |
| NadR transcriptional regulator / Nicotinamide-nucleotide adenylyltransferase, NadR family (EC 2.7.7.1) / Ribosylnicotinamide kinase (EC 2.7.1.22) | - | - | AAC77343.2 |
| Osmolarity sensory histidine kinase EnvZ | - | - | AAC76429.1 |
| Osmosensitive K+ channel histidine kinase KdpD | - | - | AAC73789.1 |
| Pantothenate kinase (EC 2.7.1.33) | - | - | AAC76952.1 |
| Phosphoribulokinase homolog, function unknown | - | - | AAC76380.1 |
| Phytochrome, two-component sensor histidine kinase (EC 2.7.3.-); Cyanobacterial phytochrome B | - | UWX65331.1 | - |
| Polyphosphate kinase (EC 2.7.4.1) | - | UWX64010.1 | AAC75554.1 |
| Putative esterase YheT functionally coupled to phosphoribulokinase homolog | - | - | AAC76378.1 |
| Putative two component system histidine kinase YedV | - | - | AAC75034.1 |
| Pyridoxal kinase (EC 2.7.1.35) | - | UWX64329.1 | AAC75471.1 |
| Pyruvate kinase (EC 2.7.1.40) | - | - | AAC74746.1 |
| Pyruvate kinase (EC 2.7.1.40) | - | - | AAC74924.1 |
| Regulator of nucleoside diphosphate kinase | - | - | AAC73711.1 |
| Rhamnulokinase (EC 2.7.1.5) | - | UWX65194.1 | AAC76886.1 |
| Ribose 1,5-bisphosphate phosphokinase PhnN (EC 2.7.4.23) | - | - | AAC77055.1 |
| Ribulokinase (EC 2.7.1.16) | - | - | AAC73174.1 |
| Ribulosamine/erythrulosamine 3-kinase potentially involved in protein deglycation | - | - | AAC74795.1 |
| Selenide,water dikinase (EC 2.7.9.3) | - | - | AAC74834.1 |
| Sensor histidine kinase BtsS | - | - | AAC75187.1 |
| Sensor histidine kinase GlrK | - | - | AAC75609.2 |
| Sensor histidine kinase PhoQ (EC 2.7.13.3) | - | - | AAC74213.1 |
| Sensor histidine kinase RcsC (EC 2.7.13.3) | - | - | AAC75278.2 |
| Sensor histidine kinase YpdA | - | - | AAC75439.1 |
| Sensory histidine kinase BaeS | - | UWX64062.1 | AAC75139.1 |
| Sensory histidine kinase CreC of two-component signal transduction system CreBC | - | - | AAC77352.1 |
| Sensory histidine kinase in two-component regulatory system with RstA | - | UWX64062.1 | AAC74681.1 |
| Sensory histidine kinase QseC | - | UWX63556.1 | AAC76062.1 |
| Shikimate kinase III (EC 2.7.1.71) | - | UWX65178.1 | AAC73491.1 |
| Signal transduction histidine kinase CheA | - | - | UMR55122.1 |
| Signal transduction histidine-protein kinase BarA (EC 2.7.13.3) | - | - | AAC75828.1 |
| Thiamine kinase (EC 2.7.1.89) @ Adenosylcobinamide kinase (EC 2.7.1.156) | - | - | AAC74190.1 |
| Thiamine-monophosphate kinase (EC 2.7.4.16) | - | - | AAC73520.1 |
| Tagatose-6-phosphate kinase GatZ (EC 2.7.1.144) | - | - | AAC75156.1 |
| Tetraacyldisaccharide 4'-kinase (EC 2.7.1.130) | - | - | AAC74001.1 |
| Thymidine kinase (EC 2.7.1.21) | - | - | AAC74320.1 |
| Thymidylate kinase (EC 2.7.4.9) | AAF09-698.1 | UWX63343.1 | AAC74182.1 |
| Toxin HigB / Protein kinase domain of HipA | - | - | AAC74580.1 |
| Transcription regulator [contains diacylglycerol kinase catalytic domain] | AAF10935.1 | UWX63071.1 | - |
| Trimethylamine-N-oxide sensor histidine kinase TorS (EC 2.7.13.3) | - | - | AAC74078.2 |
| Two-component system sensor histidine kinase | AAF12289.1 | UWX64548.1 | - |
| Two-component system sensor histidine kinase | AAF10157.1 | UWX65575.1 | - |
| Two-component system sensor histidine kinase | AAF11961.1 | UWX65580.1 | - |
| Two-component system sensor histidine kinase | AAF10322.1 | UWX65051.1 | AAC75139.1 |
| Two-component system sensor histidine kinase | AAF10470.1 | UWX65435.1 | - |
| Two-component system sensor histidine kinase | - | UWX62839.1 | - |
| Two-component system sensor histidine kinase | AAF10438.1 | UWX63217.1 | - |
| Two-component system sensor histidine kinase | AAF12539.1 | UWX63556.1 | AAC73671.1 |
| Two-component system sensor histidine kinase | AAF10322.1 | UWX63624.1 | AAC73503.1 |
| Two-component system sensor histidine kinase | AAF11119.1 | UWX63666.1 | - |
| Two-component system sensor histidine kinase | AAF10157.1 | UWX65575.1 | - |
| Two-component system sensor histidine kinase | AAF10322.1 | UWX63624.1 | - |
| Two-component system sensor histidine kinase | AAF10438.1 | UWX63217.1 | - |
| Two-component system sensor histidine kinase | AAF10470.1 | UWX65435.1 | - |
| Two-component system sensor histidine kinase | - | UWX62839.1 | - |
| Two-component system sensor histidine kinase | AAF11166.1 | UWX64693.1 | - |
| Two-component system sensor histidine kinase | AAF11875.1 | UWX63624.1 | AAC76062.1 |
| Two-component system sensor histidine kinase | AAF11961.1 | UWX65580.1 | - |
| Two-component system sensor histidine kinase | AAF12289.1 | UWX64548.1 | - |
| Two-component system sensor histidine kinase | AAF12175.1 | UWX64999.1 | - |
| Two-component system sensor histidine kinase | - | - | AAC77352.1 |
| Tyrosine-protein kinase (EC 2.7.10.2) | - | - | AAC74066.1 |
| Tyrosine-protein kinase (EC 2.7.10.2) | AAF12275.1 | UWX63104.1 | - |
| Tyrosine-protein kinase (EC 2.7.10.2) | AAF12275.1 | UWX63104.1 | - |
| Tyrosine-protein kinase (EC 2.7.10.2) => Wzc | - | - | AAC75121.2 |
| Ubiquinone biosynthesis regulatory protein kinase UbiB | - | - | AAC76838.1 |
| Uncharacterized sugar kinase YdjH | - | UWX63993.1 | AAC74842.2 |
| Uncharacterized sugar kinase YegV, PfkB family | - | - | AAC75161.1 |
| Uncharacterized sugar kinase YeiI | AAF11859.1 | UWX65101.1 | AAC75221.1 |
| Uridine kinase (EC 2.7.1.48) | AAF09747.1 | UWX64521.1 | AAC75127.2 |
| Uridylate kinase (EC 2.7.4.22) | AAF11078.1 | UWX62893.1 | AAC73282.1 |
| Uncharacterized sugar kinase YgcE (EC 2.7.1.-) | - | - | AAC75818.1 |
| Uncharacterized sugar kinase YggC | - | - | AAC75965.1 |
| YihE protein, a ser/thr kinase implicated in LPS synthesis and Cpx signalling | - | - | AAC76857.1 |
| Xylulose kinase (EC 2.7.1.17) | - | - | AAC76588.1 |

**Supplementary Table 8.** Oxidative stress defence-related proteins in *Deinococcus* *rubellus* Ant6.

| **Accession no** | **Common name** | **Description** | **COG number** |
| --- | --- | --- | --- |
| UWX65462 | SodA | Mn-containing Superoxide dismutases (SODs) | COG0605 |
| UWX63958 | KatE | monofunctional heme catalase | COG0753 |
| UWX65396 | Bcp | peroxiredoxin | COG1225 |
| UWX65550 | Bcp | peroxiredoxin | COG1225 |
| UWX64272 | AhpE | atypical type of AhpC | COG0450 |
| UWX65294 | AhpD | alkyl hydroperoxidase D-like protein (YciW) | COG2128 |
| UWX63893 | OsmC | organic hydroperoxide reductase OsmC | COG1764 |
| UWX63319 | Ohr | organic hydroperoxide reductase Ohr | COG1764 |
| UWX63295 | YhfA | uncharacterized OsmC-related protein | COG1765 |
| UWX64609 | TrxA | thioredoxin | COG0450 |
| UWX63377 | TrxB | thioredoxin-disulfide reductase | COG0492 |
| UWX65069 | MsrA | peptide methionine sulfoxide reductase MsrA | COG0225 |
| UWX63391 | MsrB | peptide methionine sulfoxide reductase MsrB | COG0229 |
| UWX63515 | HSP33 | heat shock protein 33 (HslO) | COG1281 |
| UWX65319 | GrxC | glutaredoxin | COG0695 |
| UWX65121 | grxA | glutaredoxin family protein | COG0695 |
| UWX63874 | DsbA_FrnE | DsbA family oxidoreductase | COG2761 |
| UWX65020 | DsbA-like protein | predicted dithiol-disulfide isomerase, DsbA family | COG2761 |
| UWX65077 | ExeM_NucH_DNase | ExeM/NucH family extracellular endonuclease | COG2374 |
| UWX64318 | Dps1 | DNA starvation/stationary phase protection protein | COG0783 |
| UWX65489 | MsrP | protein-methionine-sulfoxide reductase catalytic subunit | COG2041 |
| UWX65603 | MsrQ | protein-methionine-sulfoxide reductase heme-binding subunit | COG2717 |
| UWX63433 | CrtE | geranylgeranyl diphosphate synthase | COG0142 |
| UWX62945 | CrtB | phytoene synthase | COG1562 |
| UWX62946 | CrtI | phytoene desaturase | COG1233 |
| UWX64932 | CruF | carotenoid 1',2'-hydratase | COG2324 |
| N0D28_15360 | CrtD | carotenoid 3',4'-desaturase | COG1233 |
| UWX64933 | CrtO | carotenoid ketolase | COG1233 |
| UWX65635 | BshA | BSH biosynthesis glycosyltransferase | COG0438 |
| UWX63128 | BshB1 | BSH biosynthesis deacetylase | COG2120 |
| UWX62933 | BshC | BSH biosynthesis cysteine-adding enzyme | COG4365 |
| UWX65415 | YpdA | BSH reductase | COG0492 |
| UWX63582 | SufB | Fe-S cluster assembly scaffold protein | COG0719 |
| UWX63581 | SufC | Fe-S cluster assembly ATPase | COG0396 |
| UWX63584 | SufD | Fe-S cluster assembly permease | COG0719 |
| UWX65017 | SufS | cysteine desulfurase | COG1104 |
| UWX64641 | SufE | cysteine desulfuration protein | COG2166 |
| UWX64417 | SufA | Fe-S cluster assembly iron-binding protein | COG0316 |
| UWX65100 | SufU | NifU homolog involved in Fe-S cluster formation | COG0822 |
| UWX63066 | MntH | Mn2+ transporters of the NRAMP family | COG1914 |
| UWX64010 | PPK1 | polyphosphate kinase 1 | COG0855 |
| UWX63833 | PPX | exopolyphosphatase | COG1227 |
| UWX64596 | ClpP | ATP-dependent Clp protease proteolytic subunit | COG0740 |

**Supplementary Table 9: Unique domain containing proteins in *Deinococcus***

| **Proteins** | **Predicted unique domains** | | | |
| --- | --- | --- | --- | --- |
|  | **COG number** | ***D. radiodurans* R1** | ***D. rubellus* Ant6** | ***E. coli* K12** |
| RecQ protein (DR1289) | COG0514 | One recQ super family and three HRDC domains (AAF10859) | One recQ super family and two HRDC domains (UWX63339) | One recQ super family and one HRDC domain (AAT48221) |
| HRDC domain containingprotein (DR2444) | COG0210 | one HRDC, transcription termination factor Rho and AAT_I super family domain (AAF11987) | one HRDC, transcription termination factor Rho and AAT_I super family domain (UWX64634) | - |
| RecD helicase (DR1902) | COG0507 | contains a long N terminal region (AAF11453) | contains a long N terminal region (UWX63593) | N-terminal extension is missing (AAC75858) |
| Hypothetical protein(DRB0098) | COG4639 | phosphatase domain of the HD superfamily and a polynucleotide kinase domain (AAF12617) | - | - |
| Hypothetical protein (DRA0131) | COG1061 | endonuclease domain fused with additional domain RAD25-like helicase domain (AAF12227) | - | - |
| Hypothetical protein (DR1533) | COG3440 | endonuclease domain fused with additional domain SAD domain (AAF11096) | - | - |
| TerF-related protein(DRA0057) | COG1403 | endonuclease domain fused with additional domain TerDEXZ/CABP family (AAF12257) | - | - |
| ATP-dependent DNA helicase RecG-related protein (DR2199) | COG2512 | ATP-dependent DNA helicase RecG, Schlafen, AlbA_2 domain and an Winged helix DNA-binding domain (AAF11748) | Putative ATP-dependent DNA helicase recG C-terminal domain and Winged helix DNA-binding domain (UWX65593) | - |
| DEAD/DEAH box helicase (DR0065) | COG1061 | Distinct helicase family with a unique C-terminal domain including a MrfA-like Zn-binding domain (AAF09654) | Distinct helicase family with a unique C-terminal domain including a MrfA-like Zn-binding domain (UWX65430) | - |
